# Searching for SARS-COV-2 on Particulate Matter: A Possible Early Indicator of COVID-19 Epidemic Recurrence

**DOI:** 10.3390/ijerph17092986

**Published:** 2020-04-25

**Authors:** Leonardo Setti, Fabrizio Passarini, Gianluigi De Gennaro, Pierluigi Barbieri, Alberto Pallavicini, Maurizio Ruscio, Prisco Piscitelli, Annamaria Colao, Alessandro Miani

**Affiliations:** 1 Department of Industrial Chemistry, University of Bologna, 40136 Bologna, Italy; leonardo.setti@unibo.it; 2 Interdepartmental Centre for Industrial Research “Renewable Sources, Environment, Blue Growth, Energy”, University of Bologna, 47921 Rimini, Italy; fabrizio.passarini@unibo.it; 3 Department of Biology, University of Bari, 70121 Bari, Italy; gianluigi.degennaro@uniba.it; 4 Department of Chemical and Pharmaceutical Sciences, University of Trieste, 34127 Trieste, Italy; barbierp@units.it; 5 Department of Life Sciences—University of Trieste, 34127 Trieste, Italy; pallavic@units.it; 6 Division of Laboratory Medicine, University Hospital Giuliano Isontina (ASU GI), 34127 Trieste, Italy; maurizio.ruscio@asugi.sanita.fvg.it; 7 Italian Society of Environmental Medicine (SIMA), 20149 Milan, Italy; priscofreedom@hotmail.com; 8 UNESCO Chair on Health Education and Sustainable Development, University of Naples Federico II, 80131 Naples, Italy; amcolao@gmail.com; 9 Department of Environmental Sciences and Policy, University of Milan, Via Celoria 2, 20133 Milan, Italy

**Keywords:** COVID-19, RNA, Particulate Matter, Indicator, Epidemic, Relapse

## Abstract

A number of nations were forced to declare a total shutdown due to COVID-19 infection, as extreme measure to cope with dramatic impact of the pandemic, with remarkable consequences both in terms of negative health outcomes and economic loses. However, in many countries a “Phase-2” is approaching and many activities will re-open soon, although with some differences depending on the severity of the outbreak experienced and SARS-COV-2 estimated diffusion in the general population. At the present, possible relapses of the epidemic cannot be excluded until effective vaccines or immunoprophylaxis with human recombinant antibodies will be properly set up and commercialized. COVD-19-related quarantines have triggered serious social challenges, so that decision makers are concerned about the risk of wasting all the sacrifices imposed to the people in these months of quarantine. The availability of possible early predictive indicators of future epidemic relapses would be very useful for public health purposes, and could potentially prevent the suspension of entire national economic systems. On 16 March, a Position Paper launched by the Italian Society of Environmental Medicine (SIMA) hypothesized for the first time a possible link between the dramatic impact of COVID-19 outbreak in Northern Italy and the high concentrations of particulate matter (PM_10_ and PM_2.5_) that characterize this area, along with its well-known specific climatic conditions. Thereafter, a survey carried out in the U.S. by the Harvard School of Public Health suggested a strong association between increases in particulate matter concentration and mortality rates due to COVID-19. The presence of SARS-COV-2 RNA on the particulate matter of Bergamo, which is not far from Milan and represents the epicenter of the Italian epidemic, seems to confirm (at least in case of atmospheric stability and high PM concentrations, as it usually occurs in Northern Italy) that the virus can create clusters with the particles and be carried and detected on PM_10_. Although no assumptions can be made concerning the link between this first experimental finding and COVID-19 outbreak progression or severity, the presence of SARS-COV-2 RNA on PM_10_ of outdoor air samples in any city of the world could represent a potential early indicator of COVID-19 diffusion. Searching for the viral genome on particulate matter could therefore be explored among the possible strategies for adopting all the necessary preventive measures before future epidemics start.

## 1. COVID-19: An Unexpected Threat to Human Health and Society Influenced by Environmental Factors? 

A number of nations were forced to declare a total shutdown due to COVID-19 infection as an extreme measure to cope with the dramatic impact of the pandemic, with remarkable consequences in terms of negative health outcomes, social life and economic loses. This incredible choice of closing regions, States, and entire countries was seen as the only way to cope with the unexpected threat to human health represented by SARS-COV-2 virus due to the high number of infected people requiring intensive care in hospital setting in relation to the limited beds available. 

At the date of 23 April, there were about 2,700,000 confirmed cases of COVID-19 (probably an under-estimated count, due to the high proportion of asymptomatic patients that are not tested) and almost 190,000 deaths recorded worldwide. The majority of diagnoses were made both in Europe (213,000 in Spain, 190,000 in Italy, 150,000 in Germany, 140,000 in United Kingdom, and 120,000 in France) and in North America (870,000 in USA and 42,000 in Canada). In Asia, Iran (87,000) and Turkey (100,000) had more infections than China (83,850), the country where the pandemic started and was subsequently well contained in the province of Wuhan. Surprisingly, the Indian sub-continent recorded only 23,000 cases, and a minor number of cases were diagnoses in South America (50,000 in Brazil, 21,000 in Perù, and 10,000 in Chile), Africa (27,000) and Oceania (6600 in Australia and 1100 in New Zeland) [1].

The Italian population is less than 20% of that of the United States, but the number of deaths recorded in Italy (25,500) are up to 50% of those reported in the U.S. (50,000). These epidemiological figures are mainly due to the rapid spreading of COVID-19 in Lombardy and Northern Italian regions of the Po Valley (Padana Plain), an area characterized by constant high concentrations of particulate matter (PM_10_ and PM_2.5_), already recognized as one of the most polluted zones in Europe [2]. Lombardy accounts for 56% of the overall COVID-19 confirmed cases, 45% of hospital admissions and 55% of total deaths, followed by Emilia Romagna (15% of cases and 13% of deaths), Piedmont (12% of cases and 8% of deaths), and Veneto (11% of cases and 5% of deaths) [2].

The Italian Society of Environmental Medicine (SIMA) has been the first to hypothesize—by releasing a specific Position Paper on 16 March—a possible link between the high mortality rates observed in Northern Italy due to COVID-19 and the PM concentrations [3,4]. A significant correlation was found between the geographical distribution of the daily PM_10_ exceedances in 110 Italian Provinces (number of days exceeding the PM10 law limit of 50 µg/m^3^) and the spreading of the COVID-19 infection before the shutdown decided by the Government [2]. The number of PM_10_ exceedances was much more frequent in Lombardy and in cities located in the Po valley than those observed in Rome and Southern Italy, where the diffusion and lethality of the virus was significantly lower when compared to those of Northern regions [3,4]. These first observations suggest that particulate matter could be regarded—if not as a “carrier” or a “boosting factor”—at least as an indicator of the severity of COVID-19 infection in terms of diffusion and health outcomes observed in Northern Italy. At the same time, it must be pointed out that long term exposure to high levels of particulate matter may chronically impair human health and possibly influence the clinical course of infections acquired by already debilitated individuals, especially in most vulnerable age groups [5].

Meanwhile, a survey carried out in the U.S. by the Harvard School of Public Health has suggested a strong association between increases in particulate matter concentration and mortality rates due to COVID-19, with The Lancet scientific community highlighting the need for adopting a planetary health perspective on COVID-19 outbreak [6,7]. Further experimental studies could specifically assess the possibility that particulate matter may act as a “carrier” for the viral droplet nuclei, as it has been shown for other viruses, eventually impressing a “boost effect” to the spreading of the viral infection [8,9,10,11,12,13,14,15,16,17,18]. At the present, no assumptions can be made concerning the correlation between the possible presence of the virus on PM and COVID-19 outbreak progression. However, based on the available literature, there is already enough evidence to consider the airborne route, with a potential role of particulate matter, as a possible additional factor for interpreting the anomalous COVID-19 outbreaks observed in Northern Italy and in the U.S., as well as in other areas characterized by high PM concentrations [8,9,10,11,12,13,14,15,16,17,18]. On these bases, in the frame of the One Health approach that can be applied to the international emergency generated by the SARS-COV-2 as it involves the interactions between humans and environment, the Italian Society of Environmental Medicine and the UNESCO Chair on Health Education and Sustainable Development support the vision of Sir Andy Haines, who has called for the adoption of urgent actions to counteract climate changes and the alteration of ecosystems that might trigger new and unexpected threats to human health such as that of COVID-19, which we are so dramatically experiencing worldwide [19].

## 2. Approaching “Phase-2”: Searching for SARS-COV-2 on Outdoor Particulate Matter as Early Indicator of COVID-19 Epidemic Relapses?

In many countries, a “Phase-2” is approaching and many activities will re-open soon, although with some differences, depending on the severity of the outbreak experienced and SARS-COV-2 estimated diffusion in the general population. At the present, possible relapses of the epidemic cannot be excluded until effective vaccines or immunoprophylaxis with human recombinant antibodies will be properly set up and commercialized. COVD-19-related quarantines have triggered serious social challenges, so that decision makers are concerned about the risk of wasting all the sacrifices imposed to the people in these months of quarantine. The availability of possible early predictive indicators of future epidemic relapses would be very useful for public health purposes, and could potentially prevent the suspension of entire national economic systems. 

The presence of SARS-COV-2 RNA on the particulate matter of Bergamo, which is not far from Milan and represents the epicenter of the Italian epidemic, seems to confirm (at least in case of atmospheric stability and high PM concentrations, as it usually occurs in Northern Italy) that the virus can create clusters with the particles and be carried and detected on PM10 [20]. The presence of SARS-COV-2 RNA on PM10 of outdoor air samples in any city of the world could represent a potential early indicator of COVID-19 diffusion. Searching for viral genomes on particulate matter could therefore be explored among the possible strategies for adopting all the necessary preventive measures before future epidemics start. 

The availability of early direct or indirect indicators of SARS-COV-2 diffusion could save many lives and reduce the economic impact of future possible outbreaks by giving Governments enough time to cope with these new kind of challenges [21].

In Italy, lethality rates showed a remarkable increase in the oldest age groups, reaching 40% in men aged >80 years old (20% in women), and 30% in males between 70 and 79 years old (16% in females). People aged 60–69 still present a lethality rate as high as 12% in men and 5.6% in women, accounting for 15% of infections and 11% of deaths. Elderly people >80 years old represent 22% of positive cases and 50% of the deaths, while patients aged 70–79 account for additional 16% of infections and 30% of deaths [22]. These figures, along with the very high ageing index which characterizes Italy (174 elderly people aged >65 years old every 100 children aged 0–14) can raise questions about the baseline frailty of the population who has experienced the diffusion of SARS-COV-2 in such a rapid way, possibly explaining the high lethality rates observed. Actually, it could be proposed that air pollution could influence the COVID-19 outbreak progression in a direct way or also indirectly, by enhancing the host susceptibility to viral infection and independently increasing the baseline risk of cardiovascular events or complications, chronic obstructive pulmonary diseases (COPD), and other conditions that are known to increase the severity of viral infections. 

On the other hand, COVID-19 has clearly shown its potential to become a hospital infection, at least in Europe and U.S. (whose healthcare systems are usually centralized at hospital level), with 10% of infected people being healthcare workers, and a huge proportion of cases and deaths possibly related to infections acquired in hospital setting or nursing homes [23,24]. Indeed, the possibility of contagion from asymptomatic people and the epidemics disseminating within cruise ships has already demonstrated that the virus spreads very quickly in indoor environments, with this issue representing one of the most remarkable challenges during the re-opening of social activities (i.e., restaurants, offices, bars, pubs, etc.) [25,26,27]. At the present, many research groups are working on COVID-19 clinical features and on the diffusion of SARS-COV-2 in outdoor or indoor air (especially in hospital settings) as well as in water/wastewater. There is the rationale for carrying out experimental studies at European and international level, specifically aimed at confirming the presence of the SARS-CoV-2 as already demonstrated in Bergamo area [20]. An additional relevant step would consist of investigating the potential virulence of SARS-COV-2 present on particulate matter in order to verify (under prescribed condition of safety) if the virus can remain vital and infectious for a defined time on outdoor particulate matter as well as on droplet nuclei exhaled by infected people in indoor environments [28].
